# Junctional Adhesion Molecule-A Regulates Vascular Endothelial Growth Factor Receptor-2 Signaling-Dependent Mouse Corneal Wound Healing

**DOI:** 10.1371/journal.pone.0063674

**Published:** 2013-05-08

**Authors:** Sharmila Chatterjee, Yan Wang, Melinda K. Duncan, Ulhas P. Naik

**Affiliations:** 1 Department of Biological Sciences, University of Delaware, Newark, Delaware, United States of America; 2 Department Biochemistry and Chemistry, University of Delaware, Newark, Delaware, United States of America; 3 Delaware Cardiovascular Research Center, University of Delaware, Newark, Delaware, United States of America; 4 Delaware Biotechnology Institute, University of Delaware, Newark, Delaware, United States of America; Ottawa Hospital Research Institute, Canada

## Abstract

Inflammation and angiogenesis are integral parts of wound healing. However, excessive and persistent wound-induced inflammation and angiogenesis in an avascular tissue such as the cornea may be associated with scarring and visual impairment. Junctional adhesion molecule A (Jam-A) is a tight junction protein that regulates leukocyte transmigration as well as fibroblast growth factor-2 (FGF-2)-induced angiogenesis. However its function in wound-induced inflammation and angiogenesis is still unknown. In this study, we report spontaneous corneal opacity in Jam-A deficient mice associated with inflammation, angiogenesis and the presence of myofibroblasts. Since wounds and/or corneal infections cause corneal opacities, we tested the role of Jam-A in wound-induced inflammation, angiogenesis and scarring by subjecting Jam-A deficient mice to full thickness corneal wounding. Analysis of these wounds demonstrated increased inflammation, angiogenesis, and increased number of myofibroblasts thereby indicating that Jam-A regulates the wound-healing response by controlling wound-induced inflammation, angiogenesis and scarring in the cornea. These effects were not due to inflammation alone since the inflammation-induced wound-healing response in Jam-A deficient mice was similar to wild type mice. In order to determine the molecular mechanism associated with the observed aberrant corneal wound healing in Jam-A deficient mice, we assessed the expression of the components of vascular endothelial growth factor A (VEGF-A)/vascular endothelial growth factor receptor- 2(VEGFR-2) signaling pathway. Interestingly, we observed increased levels of VEGF-A mRNA in Jam-A deficient eyes. We also observed nuclear localization of phosphorylated SMAD3 (pSMAD3) indicative of TGFβ pathway activation in the Jam-A deficient eyes. Furthermore the increased wound-induced corneal inflammation, angiogenesis, and scarring in Jam-A deficient mice was attenuated by treatment with DC101, an anti-vascular endothelial growth factor receptor-2 (VEGFR-2) antibody. Our results suggest that in the absence of Jam-A, the VEGF-A/VEGFR-2 pathway is upregulated, thereby augmenting wound induced corneal inflammation, angiogenesis, and myofibroblast accumulation leading to scarring.

## Introduction

Wound healing is a dynamic process critical to restore tissue structure following damage. Wound healing is often divided into three over-lapping stages [1]. First the inflammatory stage, which results in the recruitment of macrophages and neutrophils to the site of injury [2], [3]. Macrophages help clear pathogens and cellular debris from the site of the wound. Second, the proliferative and fibrotic phase. The inflammatory cells release matrix metalloproteinases (MMPs), cytokines, and growth factors that lead to the proliferative and fibrotic stage of wound healing along with angiogenesis (also known as neovascularization) at the injured site [4], [5], [6]. This is an important step in wound healing in many tissues since these new blood vessels transport oxygen, other nutrients, and additional inflammatory cells needed for faster wound healing. Finally, at the third stage, once the wound is closed, the tissue is remodeled in an attempt to re-establish normal tissue architecture. However, myofibroblast persistence and/or excess angiogenesis can lead to scarring and compromised tissue function [7].

VEGF-A is known to promote angiogenesis during wound healing and often works in concert with cell adhesion molecules such as integrin α_v_β_6_ and integrin α_v_β_5_ in endothelial cells to mediate this function [8], [9]. VEGF-A signals by binding to its receptors VEGFR-1 (FLT-1) and VEGFR-2 and is known to also promote inflammation and epithelial to mesenchymal transition [10]. VEGF-A signaling through its receptors FLT-1 and VEGFR-2 also regulates TGFβ expression that in turn regulates corneal wound healing [11], [12], [13]. In contrast with the pro-angiogenic properties of VEGF-A and its receptors, soluble VEGFR-1 (sFLT-1) is anti-angiogenic, acting as a VEGF-A trap, which inhibits VEGF-A signaling.

Blood vessels consist of quiescent vascular endothelial cells, which form tight junctions, and maintain the vessel integrity. JAM-A is a tight junction protein that is involved in tight junction permeability [14], leukocyte transmigration [15] and FGF-2-induced angiogenesis [16]. JAM-A associates with the integrin α_v_β_3_ and is essential for FGF-2 induced endothelial cell migration on vitronectin [17], [18]. Although Jam-A deficient mice have an apparently normal vasculature, FGF-2 induced angiogenesis is defective in these mice as assayed by an *in vivo* Matrigel plug assay [16]. We observed that a significant percentage of Jam-A deficient animals on a C57Bl/6NHsd genetic background developed spontaneous corneal opacities as they age. Histological investigation of the eyes of these mice revealed increased corneal angiogenesis, inflammation and myofibroblast accumulation. We also found that Jam-A deficient mice on this inbred background have profound defects in the healing of full thickness corneal wounds due to the increased VEGF-A levels in the eye. This upregulation of VEGF-A was found to be functionally relevant since treatment of Jam-A deficient mice with a function blocking VEGFR-2 antibody resulted in a partial rescue of the observed wound-healing defect. These results suggest that in the absence of Jam-A, pathways regulating VEGF-A/VEGFR-2 signaling are upregulated, resulting in heightened inflammation, angiogenesis and scarring.

## Materials and Methods

### Animals

Generation of *Jam-A^gt/gt^* (*F11r^Gt^* ^(*pGT1pfs*)*1Upn*^/*F11r^Gt^* ^(*pGT1pfs*)*1Upn*^) mice has been previously described [16]. The mice were genotyped by the polymerase chain reaction (PCR). C57Bl/6NHsd (Harlan Laboratories), wild type (WT) and *Jam-A^gt/gt^* mice of both genders were used in this study.

### Ethical statement

This study was carried out in strict accordance with the recommendations in the Guide for the Care and Use of Laboratory Animals of the National Institutes of Health. The protocol was approved by the University of Delaware Institutional Animal Care and Use Committee (AUP no: 1094). All surgery was performed under the ketamine/xylazine anesthesia, and all efforts were made to minimize suffering.

### Antibodies

A monoclonal rat anti-mouse PECAM-1 (CD31) antibody was obtained from BD Biosciences Pharmingen (San Diego, CA; catalog # 550274), rabbit anti mouse phosphorylated VEGFR-2 (pVEGFR-2) antibody was obtained from Cell Signaling Technology (Beverly, MA; catalog # 2478), the FITC conjugated mouse monoclonal anti-α-smooth muscle actin (αSMA) antibody was obtained from Sigma (St Louis, MO; catalog # F3777), rat anti-mouse Ly-6B.2 alloantigen antibody which detects polymorphonuclear (PMN) cells was obtained from Serotec (Raleigh, NC; catalog # MCA771G) and rabbit anti-mouse MMP-9, rabbit anti-mouse collagen I, rat anti-mouse CD11b, and pSMAD3 antibodies were purchased from AbCam (Boston, MA; catalog # ab38898, ab292, ab8878, ab52903 respectively). Goat anti-rabbit Alexa Fluor 568 or donkey anti-mouse Alexa Fluor 488 labeled secondary antibodies were obtained from Life Technologies (Grand Island, NY). Draq5 was purchased from Biostatus Limited (Leicestershire, United Kingdom; catalog # DR50200). Rat anti-mouse DC101 was obtained from BioXCell (West Lebanon, NH). Rat IgG was obtained Santa Cruz Biotechnology, Inc (Santa Cruz, CA; catalog # sc2026).

### Silk suture-induced corneal angiogenesis

Silk suture-induced corneal angiogenesis assays were performed as previously described [19]. Briefly, following ketamine/xylazine anesthesia, an 8–0 silk suture was inserted into the center of the cornea of 8–10 week old mice. Erythromycin ophthalmic ointment was applied immediately after suture. The mice were observed every day for the appearance of a gross scar. The mice were sacrificed 0 hour, 3 days, 7 days, and 12 days post surgery and the corneas were analyzed by immunohistochemistry.

### Full thickness corneal wounds

Mice aged between 8–10 weeks were anesthetized with ketamine/xylazine and a full thickness wound was inflicted in the central region of the cornea using a Feather Surgical No 11 blade. The wound was closed with a 10–0 nylon suture (Ethicon) and erythromycin ophthalmic ointment was applied immediately. The mice were observed for gross signs of scarring. The mice were sacrificed at 0 hour, 3 days, 7 days, and 12 days post surgery and the cornea were analyzed by immunohistochemistry.

### Anti-VEGFR-2 (DC101) treatments

Mice aged between 8–10 weeks that underwent full thickness corneal wounds were intraperitoneally injected with 200 µg/Kg DC101 diluted in saline (DC101 concentration optimized on its ability to inhibit tumor growth in C57Bl/6NHsd mice) or rat IgG every alternate day starting from 0 hour post surgery and sacrificed on the 12^th^ day post surgery.

### Immunofluorescence

Enucleated eyes were directly embedded in optimal cutting temperature (OCT) media obtained from Sakura Finetek (Torrence, CA) without prior fixation. Cryosections (16 µm) were cut, mounted on slides, and subjected to immunofluorescence studies as described previously [20]. In brief, the sections were fixed in either in chilled acetone:methanol (1∶1) for 20 minutes or paraformaldehyde and 0.25% Triton X-100 and then blocked in 3% bovine serum albumin (BSA) in phosphate buffered saline (PBS) (blocking buffer) for 1 hour at room temperature (RT). Anti-pSMAD3 stained sections were blocked in 5% BSA with goat and horse serum for 1 hour at RT. The sections were then incubated with appropriate primary antibodies diluted in blocking buffer at 4°C overnight. The slides were washed and the primary antibodies detected by incubation with the appropriate secondary antibodies, diluted in blocking buffer along with a (1∶1000) dilution of the DNA specific nucleic acid stain, Draq5 to detect the cell nucleus. The sections were analyzed and imaged using either a Zeiss LSM 5LIVE High-Speed or a Zeiss LSM 510 confocal microscope. Immunofluorescence quantitation was performed using Volocity 5.2 software (Perkin Elmer Boston, MA). A ratio of the area occupied by the individual immunostain to the total corneal area in each image was determined.

### Quantitative Real Time Polymerase Chain Reaction (Q-rtPCR)

To perform Q-rtPCR, RNA from whole eyes except for the lens of 32–48 week old WT and *Jam-A^gt/gt^* mice were used. The RNA was isolated using the RNeasy kit obtained from Qiagen (Valencia, CA). One μg of RNA was used from both WT and *Jam-A^gt/gt^* mice to prepare cDNA using the high capacity cDNA reverse transcription kit obtained from Applied Biosystems (Foster city, CA). The primers used for Q-rtPCR are shown in Table-1. Quantitative analysis of mRNA expression was performed using ABI Prism 7300 and SYBR green. Data is presented as a fold increase compared to WT values (2∧-ΔΔct) relative to β-Actin expression.

**Table 1:** Primer sequences used for Q-rtPCR amplification.

VEGF-A FWD	5′ AAGGAGGAGGGCAGAATCAT3′
VEGF-A REV	5′ ATCTGCATGGTGATGTTGGA3′
β-ACTIN FWD	5′-GCCTTCCTTCTTGGGTATGG-3′
β-ACTIN REV	5′-ACGCAGCTCAGTAACAGTCC-3′;
FLT-1 FWD	5′-TTCGGAAGACAGAAGTTCTCGTT-3′
FLT-1 REV	5′-GACCTCGTAGTCACTGAGGTTTTG-3
sFLT-1 FWD	5′-GGGAAGACATCCTTCGGAAGA-3′
sFLT-1 REV	5′-TCCGAGAGAAAATGGCCTTTT-3′
VEGFR-2 FWD	5′-GACTGTGGCGAAGTGTTTTTGA-3′
VEGFR-2 REV	5′-GTGCAGGGGAGGGTTGGCGTAG-3

### Quantitation and statistical analysis

Statistical analysis of the data was performed using Student's t-test (mean ±SEM value; SEM, standard error of the mean). P<0.05 is regarded as statistically significant at a 95% confidence interval.

## Results

### *Jam-A^gt/gt^* eyes with spontaneous corneal opacity exhibit inflammation, angiogenesis and scarring

**Figure 1:**
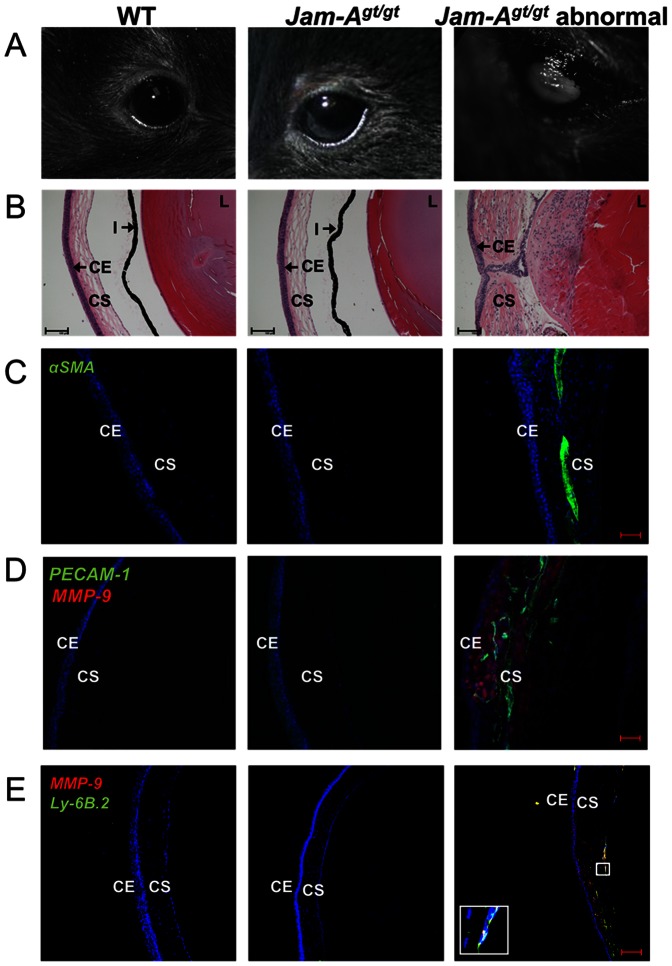
*Jam-A^gt/gt^* mice reveal corneal scarring. Representative images of whole eye and sections of WT, *Jam-A^gt/gt^* “normal” and *Jam-A^gt/gt^* “abnormal” eye of ∼32 weeks old mice. (A) Photographic images of eyes show a white corneal haze develop in some *Jam-A^gt/gt^* mice. (B) H&E staining show morphological differences between *Jam-A^gt/gt^*, WT and *Jam-A^gt/gt^* abnormal eyes scale bar 100 µm. (C-E) Confocal images of eye sections. (C) Corneal scarring shown by αSMA expression is indicative of presence of myofibroblasts. Scale bar 50 µm. (D) Corneal angiogenesis and inflammation depicted by PECAM-1 and MMP-9 staining. Scale bar 50 µm. (E) Co-immunostaining of eye sections with anti-MMP-9 and anti-Ly-6B.2 antibodies. Scale bar 100 µm. Inset magnification is 10x of the highlighted region. Magnified region shows co-localization of MMP-9, Draq5 and Ly-6B.2 which appears white. Nuclear staining using Draq5 is shown in blue. CE: Corneal epithelium; CS: Corneal stroma; L: Lens; I: Iris.

We have previously reported that the corneas of *Jam-A^gt/gt^* (*F11r^Gt^* ^(*pGT1pfs*)*1Upn*^/*F11r^Gt^* ^(*pGT1pfs*)*1Upn*^) mice are transparent and exhibit no differences in corneal epithelial debridement wound healing compared to wild type mice (WT) [21]. Since these animals were on a mixed C57Bl/6-129 background (B6.129P2-*F11r^Gt^* ^(*pGT1pfs*)*1Upn*^), we backcrossed them to C57Bl/6NHsd for 10 generations to move the *F11r^Gt^* ^(*pGT1pfs*)*1Upn*^ allele to an inbred background. We have observed that approximately 16% of congenic C57Bl/6NHsd *Jam-A^gt/gt^* mice develop spontaneous corneal opacities (Fig. 1A) with an incidence that increases with age, while this phenotype was never observed in strain matched WT controls (Table 2). Histological analysis of the paraffin embedded eyes of these mice revealed that the opaque corneas exhibited thickening and increased cellularity of the corneal stroma and disorganization of the corneal epithelial layer. However this phenotype was absent in both WT mice of the same strain and *Jam-A^gt/gt^* mice with transparent corneas (Fig. 1B). The eyes of affected *Jam-A^gt/gt^* mice also often exhibited anterior subcapsular cataracts typified by a multilayered lens epithelium (Fig. 1B). The corneal opacities of *Jam-A^gt/gt^* mice could be attributed to a scarring response since some cells of the corneal stroma were found to express αSMA, indicative of the presence of myofibroblasts, which are absent from the normal cornea (Fig. 1C). Furthermore, the opaque corneas from *Jam-A^gt/gt^* mice exhibited enhanced neovascularization (as measured by PECAM-1 expression). There was also a robust upregulation of MMP-9 expression (Fig. 1D), which is known to be expressed in corneas exhibiting scarring. The enhanced MMP-9 expression in scarred corneas is due to the infiltration of inflammatory cells [22], [23]. The upregulated MMP-9 expression observed in the abnormal *Jam-A^gt/gt^* eyes co-localized with Ly-6B.2 staining (Fig. 1E) and not αSMA (Fig. S1), indicating that the source of MMP-9 was from the inflammatory cells such as neutrophils and not myofibroblasts. However since all of the MMP-9 expressing cells did not co-localize with Ly-6B.2 expressing cells, we believe that some of the MMP-9 expression observed could be by the other inflammatory cells such as macrophages [24].

**Table 2:** Incidence of scarring in *Jam-A ^gt/gt^* and WT mice.

Age	*Jam-A ^gt/gt^*	WT
0–3 months	6/30	0/36
3–6 months	4/72	0/50
6–12 months	9/17	0/9

### *Jam-A^gt/gt^* mice show increased wound-induced corneal angiogenesis

**Figure 2:**
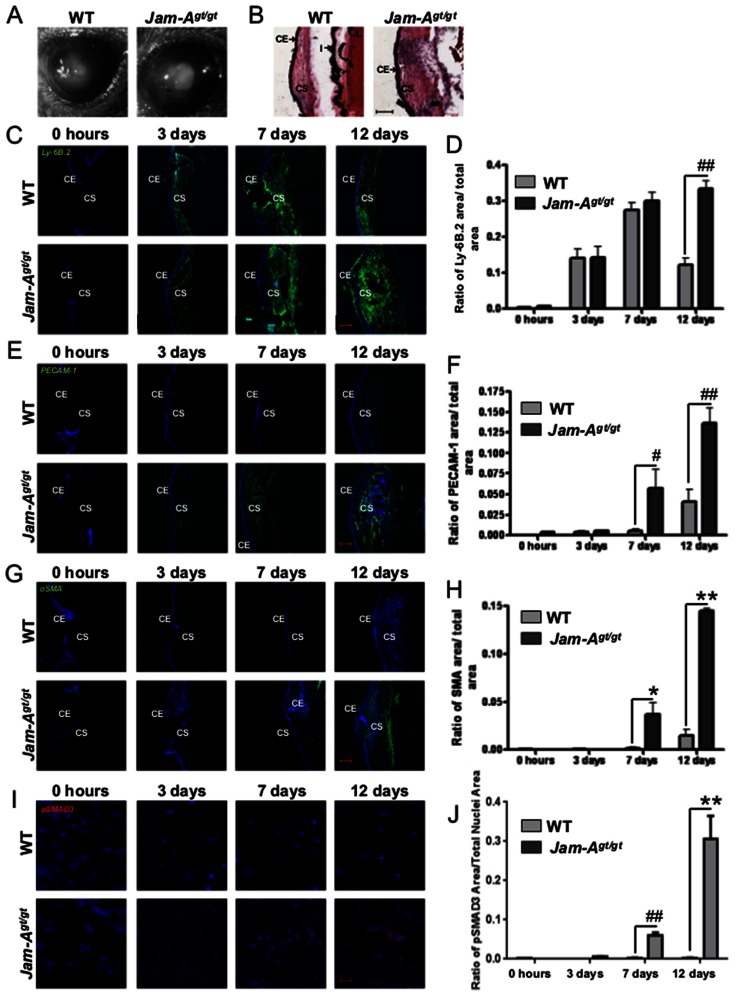
Heightened wound-induced corneal angiogenesis in *Jam-A^gt/gt^* mice. (A) Representative photographic images of corneal haze in WT and *Jam-A^gt/gt^* mice 12 days post full thickness corneal injury. (B) H&E staining of the *Jam-A^gt/gt^* and WT eyes 12 days post injury embedded in OCT show morphological differences. (C,E,G,&I) Confocal images of WT and *Jam-A^gt/gt^* eye sections at 0 hours, 3 days, 7 days, and 12 days post full thickness corneal injury. Scale bar 100 µm except for (I), which is 10 µm. Nuclear staining using Draq5 is shown in blue. (C) Ly-6B.2 staining depicts inflammation. (D) Quantitation of Ly-6B.2 staining in the *Jam-A^gt/gt^* and WT mice is shown by the ratio of the area covered by Ly-6B.2 to the total corneal area in each image. (E) Angiogenesis demonstrated by PECAM-1 staining. (F) The quantitation of PECAM-1 staining in the *Jam-A^gt/gt^* and WT mice is shown by the ratio of the area covered by PECAM-1 to the total corneal area in each image. (G) αSMA expression indicating keratocyte activation leading to myofibroblast formation in *Jam-A^gt/gt^* and WT mice. (H) Quantitation of αSMA expression in the *Jam-A^gt/gt^* and WT corneas. (I) pSMAD3 staining indicative of TGFβ activation is shown. (J) Quantitation of pSMAD3 levels shown as a ratio of pSMAD3 stained area to the total nuclear area. The number of *Jam-A^gt/gt^*, WT mice used: 0 hours (n = 3); Day 3 (n = 3); Day 7 (n = 4); Day 12 (n = 6). (*P<0.05; **P<0.0005; #P<0.005; ##P<0.0001). CE: Corneal epithelium; CS: Corneal stroma; L: Lens; I: Iris.

Corneal wound healing has been studied by inducing corneal trauma by various methods such as burns, the placement of corneal sutures, epithelial scrape wounds, and full thickness wounds [19], [21], [25], [26]. Corneal scarring is often observed in response to a corneal injury or inflammation to the cornea [19], [27]. A full thickness corneal wound causes a break in the Descemet's membrane leading to extracellular matrix (ECM) remodeling [28]. This is associated with inflammation as well as neovascularization [29], [30]. Since our data suggested that *Jam-A^gt/gt^* mice were inappropriately sensitized to spontaneous corneal scarring, we next tested their ability to heal full thickness corneal wounds. In both WT and *Jam-A^gt/gt^* mice, corneal transparency was compromised by the 12^th^ day post injury; however, *Jam-A^gt/gt^* corneas were more severely affected than controls (Fig. 2A). Hematoxylin and eosin (H&E) staining showed that injured *Jam-A^gt/gt^* corneas appeared more abnormal by the 12^th^ day post injury than WT controls with an increased corneal thickness and stromal cellularity (Fig. 2B). Full thickness wounds caused a significant (p<0.0001) increase in inflammation as assessed by PMN accumulation in the *Jam-A^gt/gt^* corneas by the 12^th^ day post wounding compared to the WT corneas (Fig. 2C&D). This data was further supported by a similar increase in MMP-9 and CD11b (a marker for neutrophils) [31] expression observed in the *Jam-A^gt/gt^* corneas by the 12^th^ day post wounding compared to the WT corneas (Fig. S2A). MMP-9 staining pattern was found to co-localize with CD11b staining indicating that the MMP-9 expressing cells also express CD11b (Fig. S2A&B). Co-localization of Ly-6B.2 with MMP-9 confirmed that MMP-9 expressing cells are PMN. Since the MMP-9 expressing cells express both Ly-6B.2 as well as CD11b, these observations collectively suggest that these inflammatory cells are neutrophils (Fig. S2C&D). *Jam-A^gt/gt^* corneas also exhibited significantly (p<0.0001) more neovascularization than controls by the seventh day post injury as measured by the extent of PECAM-1 staining (Fig. 2E&F). Wounded *Jam-A^gt/gt^* corneas also demonstrated upregulated myofibroblast accumulation as shown by αSMA expression by the seventh day post injury, which continued to increase until at least the 12th day post injury, while injured WT corneas exhibited limited αSMA expression (Fig. 2G). Quantification revealed a significant (p<0.0005) difference in αSMA expression between *Jam-A^gt/gt^* and WT mice (Fig. 2H). Along with the increased αSMA expression observed, the wounded *Jam-A^gt/gt^* corneas also exhibited extensive collagen I deposition indicative of fibrosis (Fig S3). αSMA is expressed in both pericytes as well as myofibroblasts [32], [33]. To determine the source of αSMA expression, we performed co-immunostaining experiments with anti-αSMA and PECAM-1 antibodies. We found minimal αSMA expression in close proximity to PECAM-1 expression suggesting that pericytes were not the major source of αSMA (Fig. S4A). Since TGFβ activation leads to expression of αSMA [34], we investigated if the TGFβ pathway was activated in the *Jam-A^gt/gt^* eyes. We observed nuclear localization of pSMAD3 indicative of TGFβ activation in the *Jam-A^gt/gt^* corneas that was not observed in WT corneas (Fig. 2I&J). These abnormalities associated with the excessive wound-healing response in the *Jam-A^gt/gt^* cornea indicate that, in WT mice, Jam-A negatively regulates wound-induced inflammation, angiogenesis as well as myofibroblast accumulation in the cornea following full thickness wounds. However, we observed no difference in Jam-A expression in the WT eyes at different time points post injury suggesting that Jam-A is not upregulated as a result of wounding (Fig. S5A).

### No difference in inflammation-induced corneal angiogenesis between *Jam-A^gt/gt^* and WT eyes

**Figure 3:**
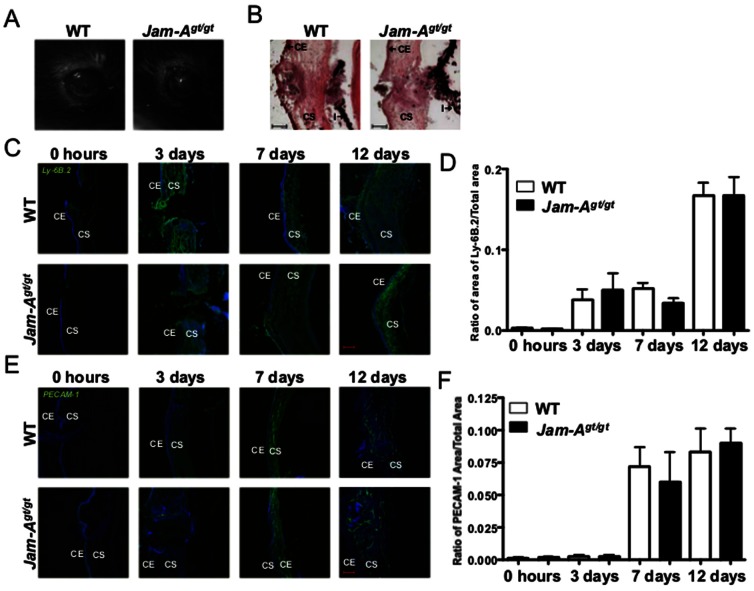
No difference in inflammation induced corneal angiogenesis between *Jam-A^gt/gt^* and WT mice. (A) Photographic images of WT and *Jam-A^gt/gt^* eyes. (B) H&E staining of frozen sections prepared from *Jam-A^gt/gt^* and WT eyes 12 days post suture. (C&E) Confocal images. Nuclear staining using Draq5 is shown in blue. (C) Corneal inflammation demonstrated by Ly-6B.2 staining. (D) Quantification of Ly-6B.2 staining shows no difference in inflammation between *Jam-A^gt/gt^* and WT mice. (E) Angiogenesis depicted by PECAM-1 staining in *Jam-A^gt/gt^* and WT mice. (F) Quantification of PECAM-1 staining from E. The number of *Jam-A^gt/gt^* and WT mice used: 0 hours (n = 3); Day 3 (n = 3); Day 7 (n = 5); Day 12 (n = 6). There was no significant difference observed in inflammation or angiogenesis between the *Jam-A^gt/gt^* and WT mice. Scale bar 100 µm. CE: Corneal epithelium; CS: Corneal stroma; I: Iris.

Inflammation is known to induce angiogenesis in a variety of tissues including cornea [19], [35]. Silk suture placement is routinely used to study inflammation induced corneal angiogenesis since silk proteins induce a foreign body immune response [19], [36], [37]. Since *Jam-A^gt/gt^* corneas showed an increased inflammatory response in response to full thickness corneal injury than WT mice (Fig. 2G&H), we next tested whether inflammation itself was sufficient to induce the enhanced angiogenesis and myofibroblast accumulation observed in injured *Jam-A^gt/gt^* corneas. We induced inflammation using the silk suture model of corneal injury by introducing silk suture into the stroma of the cornea without disturbing the Descemet's membrane (Descemet's membrane is broken in the full thickness wounds). First, in both WT and *Jam-A^gt/gt^* mice, placement of a central silk suture in the cornea did not produce overt corneal opacities by the 12th day post injury (Fig. 3A). This was supported by H&E staining of cryosections, which did not reveal any obvious morphological differences between *Jam-A^gt/gt^* and WT corneas (Fig. 3B). Also the *Jam-A^gt/gt^* mice did not show increased stromal cellularity upon introduction of a silk suture in contrast to what was observed in *Jam-A^gt/gt^* mice after full thickness corneal wounding (Fig. 2B). As expected, the silk suture did induce extensive corneal inflammation as measured by elevated Ly-6B.2 staining in both *Jam-A^gt/gt^* and WT corneas (Fig. 3C). However, there was no difference in Ly-6B.2 staining observed between *Jam-A^gt/gt^* and WT corneas (Fig 3D). Furthermore, the extent of silk suture-induced neovascularization was similar between *Jam-A^gt/gt^* and WT corneas (Fig. 3E&F). Interestingly, myofibroblasts and fibrosis were both absent as indicated by a lack of αSMA and collagen I staining at any time investigated after suture placement respectively (data not shown). There was also no difference found in the expression of Jam-A in the corneas of the WT mice at different time points post silk suture placement (Fig. S5B). These results suggest that inflammation induced angiogenesis is not the cause for the wound-induced opacity observed in the *Jam-A^gt/gt^* corneas.

### Enhanced wound-induced inflammation, angiogenesis and scarring are dependent on VEGFR-2 signaling

**Figure 4:**
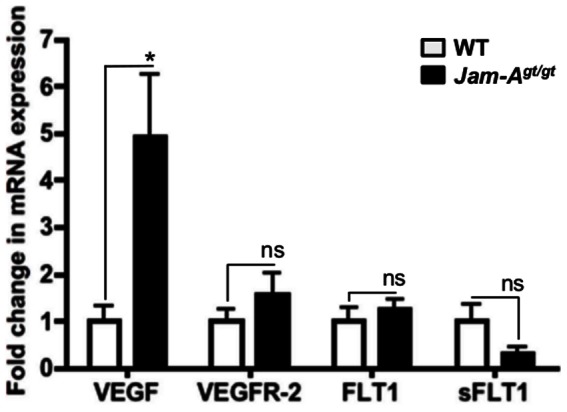
Increased VEGF-A expression in *Jam-A^gt/gt^* eyes. mRNA expression was analyzed in *Jam-A^gt/gt^* and WT mice by Q-rtPCR using whole eye tissue minus the lens. mRNA expression was quantified for VEGF-A, VEGFR-2, FLT-1 and sFLT-1 (n = 4) (*P = 0.03) (ns: not significant).

Since VEGF-A signaling is known to be involved in inflammation, neovascularization as well as accumulation of myofibroblasts, we then investigated whether the VEGF-A/VEGFR-2 pathway [38] is upregulated in *Jam-A^gt/gt^* mice [39]. Q-rtPCR analysis of RNA isolated from *Jam-A^gt/gt^* and WT eyes indicated that VEGF-A mRNA levels were significantly (P = 0.03) elevated in *Jam-A^gt/gt^* eyes (Fig. 4). Further, the mRNA levels of FLT1 (VEGFR-1) or VEGFR-2 appeared to be slightly increased in *Jam-A^gt/gt^* compared to WT mice although these differences were not statistically significant (Fig. 4). The mRNA levels of sFLT-1, which is a soluble form of VEGFR-1 recently shown to regulate corneal avascularity by acting as a VEGF-A trap [40], was also apparently downregulated in *Jam-A^gt/gt^* eyes compared to the controls, however the difference also did not reach significance at 95% confidence.

**Figure 5:**
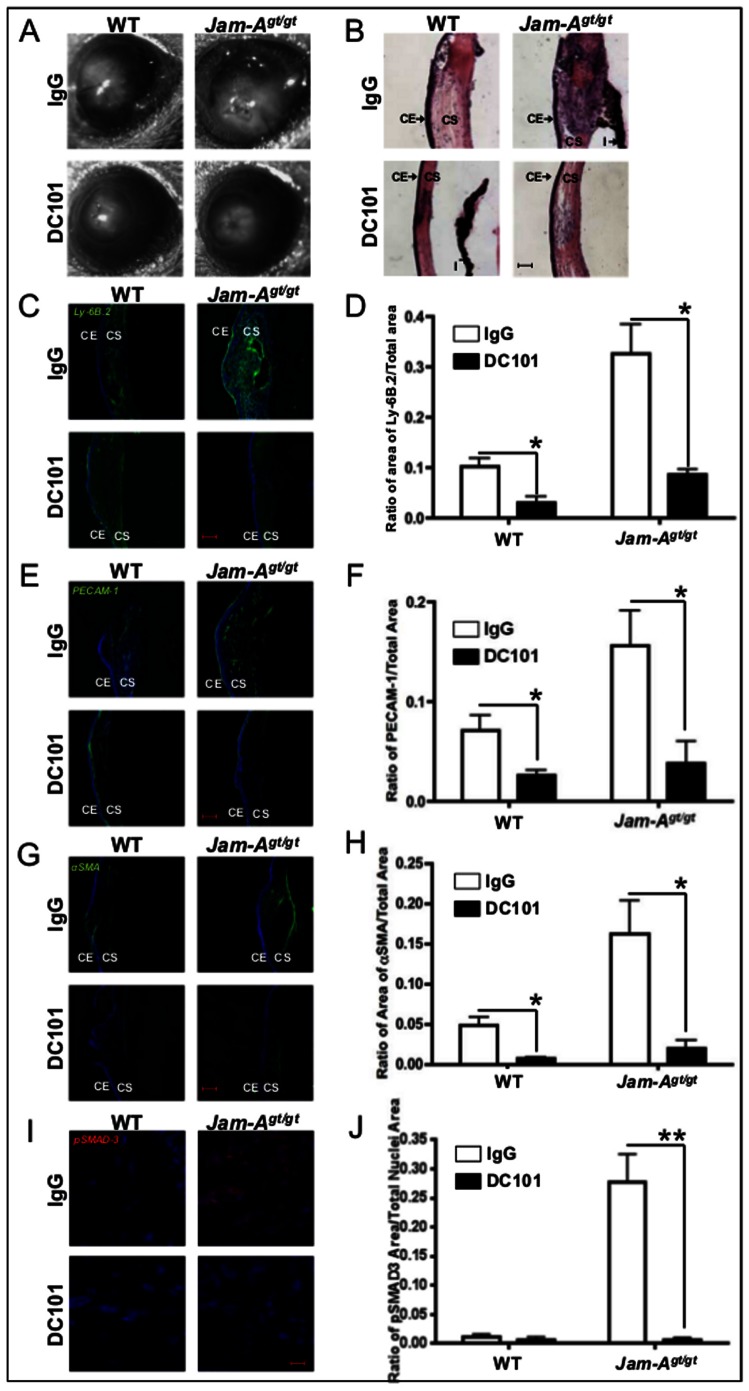
*Jam-A^gt/gt^* eyes reveal increased VEGFR-2 signaling during wound-induced corneal angiogenesis. (A) Photographic images of WT and *Jam-A^gt/gt^* mice exhibiting corneal scar observed 12 days post corneal injury and DC101 treatment. (B) H&E staining of OCT embedded eyes of *Jam-A^gt/gt^* and WT mouse treated with IgG and DC101 demonstrate corneal morphology 12 days post wound. (C, E, G, &I) Confocal images of eye sections of IgG and DC101 treated WT and *Jam-A^gt/gt^* mice. Scale bar 100 µm except for (I), which is 10 µm. Nuclear staining using Draq5 is shown in blue. (C) Ly-6B.2 staining showed inflammation in *Jam-A^gt/gt^* and WT mice. (D) Quantitation of Ly-6B.2 staining of C. (E) PECAM-1 staining to indicate angiogenesis. (F) Quantitation of PECAM-1 levels upon DC101 treatment compared to IgG control. (G) αSMA expression indicative of presence of myofibroblasts in *Jam-A^gt/gt^* and WT mice. (H) Quantitation of αSMA staining in G. (I) pSMAD3 staining indicative of TGFβ activation in IgG and DC101 treated eyes. (J) Quantification of pSMAD3 staining shown as a ratio of area of pSMAD3 to the total area occupied by nuclei. (n = 3) (*P<0.05,**P<0.0001). CE: Corneal epithelium; CS: Corneal stroma; I: Iris.

In order to test whether the increase in VEGF-A expression in *Jam-A^gt/gt^* eyes could cause the dysregulation of corneal wound healing in *Jam-A^gt/gt^* mice by signaling through VEGFR-2, we first determined activation of VEGFR-2 as assessed by pVEGFR-2 staining in the full thickness injury WT and *Jam-A^gt/gt^* corneas. We observed minimal pVEGFR-2 staining in the WT corneas. However, extensive pVEGFR2 staining was observed in the corneal epithelium of the *Jam-A^gt/gt^* eyes 12 days post injury with some staining in the stromal cells (Fig. S6). We also blocked VEGFR-2 signaling by treating the mice post injury with DC101, which is known to block VEGFR-2 function [41]. Treatment with DC101 reduced corneal opacity following full thickness corneal injury as compared to IgG treated mice (Fig. 5A). H&E staining showed that DC101 treatment also attenuated the increase in corneal thickness and stromal cellularity compared to IgG treated *Jam-A^gt/gt^* mice 12 days after full thickness corneal wounds (Fig. 5B). DC101 treatment was also associated with a down regulation in the number of PMN cells in the corneal stroma (Fig. 5C&D). We also observed a significant decrease in neovascularization as measured by the extent of PECAM-1 staining upon DC101 treatment in both WT and *Jam-A^gt/gt^* corneas (Fig. 5E&F). DC101 treatment also inhibited the accumulation of myofibroblasts following full thickness corneal injury of *Jam-A^gt/gt^* and WT mice (Fig. 5G&H). The presence of pericytes was also diminished in these corneas as shown by the reduced expression of αSMA that is associated with PECAM-1 (Fig. S4B). We further observed the absence of TGFβ activation in the DC101 treated *Jam-A^gt/gt^* eyes as compared to the IgG treated *Jam-A^gt/gt^* eyes as indicated by the lack of nuclear pSMAD3 staining (Fig. 5I&J). The above results show that JAM-A negatively regulates wound-induced inflammation, angiogenesis and scarring by modulating the VEGF-A/VEGFR-2 pathway.

## Discussion

The process of wound healing involves inflammation, neovascularization, and tissue remodeling. We have observed that a significant percentage of *Jam-A^gt/gt^* mice develop spontaneous corneal scarring, inflammation, and angiogenesis. This was mimicked in experimentally-induced full thickness corneal wounds in *Jam-A^gt/gt^* mice. We observed that this corneal scarring phenotype is not due to inflammation alone and requires TGFβ activation. Finally, we show that the resulting increased VEGF-A dependent VEGFR-2 signaling contributes to the observed propensity for corneal scarring in Jam*-A^gt/gt^* mice. This demonstrates a novel function for Jam-A in modulating the wound-healing response.

Corneal trauma such as a full thickness wound disturbs the stromal architecture and induces ECM remodeling as the stromal keratocytes are further primed to attain a myofibroblast-like phenotype [28] [42] [43]. Mechanistically, corneal scarring is believed to be caused by secretion of TGFβ2 by corneal epithelial cells into the underlying stroma, activating stromal keratocytes to undergo a myofibroblast-like transformation [32], [44]. This TGFβ-induced myofibroblast-like transformation is stabilized by topographic cues provided by the surrounding extracellular matrix (ECM). Consistent with this, we observed that introduction of silk suture does not cause corneal scarring despite the prevalence of inflammation and neovascularization. The increased Ly-6B.2 staining in the silk sutured *Jam-A^gt/gt^* corneas at a later time point could be due to the inability of the *Jam-A^gt/gt^* neutrophils to egress since JAM-A has been shown to be involved in neutrophil transmigration [15]. Along with corneal wounds or inflammation, corneal scarring can also occur in the absence of an external agent as observed in congenital stromal dystrophy caused by mutations in the gene for decorin. In this case, loss of decorin yields a loss of corneal transparency due to inappropriate activation of the TGFβ pathway leading to myofibroblast accumulation [45], [46], [47], [48].

VEGF-A is required for the process of wound healing [49] however excess VEGF-A/VEGFR-2 signaling can cause inappropriate wound healing responses that leads to scarring [50]. While VEGF-A/VEGFR-2 signaling is often thought to mostly regulate angiogenesis [51], [52], it also contributes to recruitment of inflammatory cells leading to inflammation [53]. Further, VEGF-A induced signals have been shown to induce expression of αSMA in cancer cells [54] which could explain the scarring phenotype observed in the Jam-A deficient corneas due to the inappropriate accumulation of myofibroblasts. In the case of *Jam-A^gt/gt^* mice, the increased VEGF-A could further increase the activity of the TGFβ pathway resulting in the heightened scarring phenotype observed in the Jam-A deficient mice. This is consistent with the observed positive feedback loop between VEGF-A and TGFβ [10], [12] that contributes to VEGF-A dependent scarring.

VEGF-A/VEGFR-2 expression is modulated by FGF-2 signaling in blood vessel endothelial cells (BVECs) [55], [56]. It is possible that the increased VEGF-A/VEGFR-2 signaling in the Jam-A deficient mice is due to a compensatory mechanism for the loss of FGF-2 signaling. However VEGFR-2 expression is reduced in the absence of FGF-2 signaling, while an increase in FGF-2 signaling causes increased VEGF-A signaling in BVECs [55], [56] ruling out the possibility of a compensatory mechanism. It is known that integrin α_v_β_3_ regulates NFκβ dependent VEGF-A expression in BVECs [33]. It is also known that integrin α_v_β_3_ associates with JAM-A in unstimulated BVECs and dissociates upon FGF-2 stimulation [57]. It can be speculated that JAM-A associated with integrin α_v_β_3_ keeps the integrin in an inactive conformation thereby keeping VEGF-A expression by BVECs in check. Alternatively, since JAM-A and VEGF-A are expressed in many ocular cell types besides endothelial cells [14], [58], JAM-A may be regulating VEGF-A expression in the eye via as yet unknown mechanisms.

**Figure 6:**
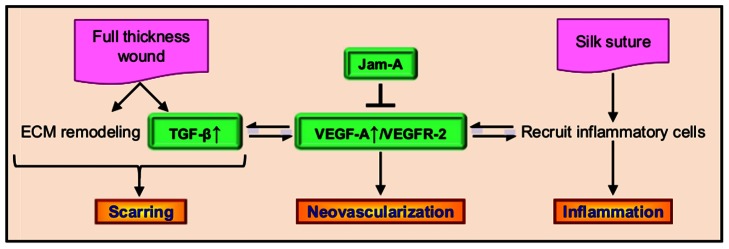
Proposed mechanism by which Jam-A regulates corneal inflammation, neovascularization, and scarring. Jam-A negatively governs VEGF-A expression thereby regulating VEGFR-2 signaling pathway. The VEGF-A/VEGFR-2 pathway leads to angiogenesis as well as recruits inflammatory cells. The VEGF-A/VEGFR-2 pathway also has a positive feed back loop with TGFβ pathway that along with ECM remodeling leads to scarring. Introduction of silk suture results in inflammation leading to neovascularization and no scarring due to the presence of an intact Descemet's membrane. Full thickness wounds that cause a break in the Descemet's membrane lead to ECM remodeling and TGFβ pathway activation that together can lead to scarring. Jam-A deficient mice exhibit increased VEGF-A/VEGFR-2 signaling and demonstrate increased inflammation, angiogenesis as well as scarring compared to WT mice.

Based on the data presented in this paper, we propose a model (Fig. 6) for the role of JAM-A in corneal wound healing. JAM-A suppresses VEGF-A expression in the cornea. In a full thickness wound, there is ECM remodeling and TGFβ signaling in addition to inflammation and neovascularization leading to corneal scarring. Introduction of a silk suture results in corneal inflammation and neovascularization, but no scarring due to the intact Descemet's membrane. All these events are caused by increased VEGF-A/VEGFR-2 signaling, since blocking VEGFR-2 signaling after full thickness corneal wounding attenuated all the three processes associated with wound healing. These observations are consistent with previously published effects of VEGF-A [51], [53]. In summary, here we demonstrate that Jam-A can control basal VEGF-A expression levels in the eye. Further, we show that this control of VEGF-A expression is important to regulate the appropriate levels of wound-induced inflammation and angiogenesis for optimum healing of full thickness corneal wounds. However, further investigation is needed to understand the mechanisms by which Jam-A regulates basal levels of VEGF-A expression levels in the eye.

## Supporting Information

Figure S1 Source of MMP-9 expression in the cornea *Jam-A^gt/gt^* mice is not from αSMA expressing cells. Confocal images of co-immunostaining with MMP-9 and αSMA antibodies in WT, *Jam-A^gt/gt^* and *Jam-A^gt/gt^* abnormal eyes. Scale bar 100 µm. Inset magnifications are 10x of the highlighted region. Nuclear staining using Draq5 is shown in blue. CE: Corneal epithelium; CS: Corneal stroma.
(TIF)

Figure S2 Inflammation observed in full thickness wounds is caused by neutrophils. (A–D) Confocal images of WT and *Jam-A^gt/gt^* eye sections. (A) Co-immunostaining with MMP-9 and CD11b antibodies show complete co-localization. Scale bar 100 µm. (B) Split representative images of MMP-9 and CD11b staining of 12 day WT eyes are also shown. Scale bar 10 µm (C) Co-localization of MMP-9 and Ly-6B.2 depicted in WT and *Jam-A^gt/gt^* corneas of full thickness injury eyes. Scale bar 100 µm. (D) Split images of MMP-9 and Ly-6B.2 staining of 12 day WT are also shown. Scale bar 10 µm. All inset magnifications are 10x of the highlighted region. Nuclear staining using Draq5 is shown in blue. CE: Corneal epithelium; CS: Corneal stroma.
(TIF)

Figure S3 Full thickness *Jam-A^gt/gt^* eyes reveal fibrosis. Collagen I and αSMA expression indicative of fibrosis depicted in full thickness WT and *Jam-A^gt/gt^* corneas. Scale bar 100 µm. Inset magnifications are 10x of the highlighted region. Nuclear staining using Draq5 is shown in blue. CE: Corneal epithelium; CS: Corneal stroma.
(TIF)

Figure S4 Pericyetes are not the major source of αSMA in full thickness wounds. (A) Full thickness wounds show minimal co-localization between PECAM-1 and αSMA staining. (B) Confocal images of co-immunostaining with PECAM-1 and αSMA antibodies in DC101 and IgG treated full thickness injury corneas. Scale bar 100 µm. All inset magnifications are 10x of the highlighted region. Nuclear staining using Draq5 is shown in blue. CE: Corneal epithelium; CS: Corneal stroma.
(TIF)

Figure S5 No difference in Jam-A expression in WT eyes during wound healing. (A) Jam-A staining of WT full thickness injury corneas at different time points post surgery. (B) Jam-A staining of WT silk sutured corneas. Scale bar 100 µm. Nuclear staining using Draq5 is shown in blue. CE: Corneal epithelium; CS: Corneal stroma.
(TIF)

Figure S6 Full thickness wounds show VEGFR-2 activation in *Jam-A^gt/gt^* corneas. pVEGFR-2 staining in *Jam-A^gt/gt^* and WT eyes in full thickness injury model. Scale bar 10 µm. Nuclear staining using Draq5 is shown in blue. CE: Corneal epithelium; CS: Corneal stroma.
(TIF)

## References

[1] Fini ME (1999) Keratocyte and fibroblast phenotypes in the repairing cornea. Prog Retin Eye Res 18: 529–551. 10217482 10.1016/s1350-9462(98)00033-0

[2] Phillipson M, Kubes P (2011) The neutrophil in vascular inflammation. Nat Med 17: 1381–1390. 22064428 10.1038/nm.2514 PMC7095830

[3] Wilson K (1997) Wound healing: the role of macrophages. Nurs Crit Care 2: 291–296. 9887766

[4] Ardi VC, Kupriyanova TA, Deryugina EI, Quigley JP (2007) Human neutrophils uniquely release TIMP-free MMP-9 to provide a potent catalytic stimulator of angiogenesis. Proc Natl Acad Sci U S A 104: 20262–20267. 18077379 10.1073/pnas.0706438104 PMC2154419

[5] Gong Y, Koh DR (2010) Neutrophils promote inflammatory angiogenesis via release of preformed VEGF in an in vivo corneal model. Cell Tissue Res 339: 437–448. 20012648 10.1007/s00441-009-0908-5

[6] Retini C, Vecchiarelli A, Monari C, Tascini C, Bistoni F, et al (1996) Capsular polysaccharide of Cryptococcus neoformans induces proinflammatory cytokine release by human neutrophils. Infect Immun 64: 2897–2903. 8757810 10.1128/iai.64.8.2897-2903.1996 PMC174164

[7] Whitcher JP, Srinivasan M, Upadhyay MP (2001) Corneal blindness: a global perspective. Bull World Health Organ 79: 214–221. 11285665 PMC2566379

[8] Hood JD, Frausto R, Kiosses WB, Schwartz MA, Cheresh DA (2003) Differential alphav integrin-mediated Ras-ERK signaling during two pathways of angiogenesis. J Cell Biol 162: 933–943. 12952943 10.1083/jcb.200304105 PMC2172815

[9] Blanco-Mezquita JT, Hutcheon AE, Stepp MA, Zieske JD (2011) alphaVbeta6 integrin promotes corneal wound healing. Invest Ophthalmol Vis Sci 52: 8505–8513. 21960555 10.1167/iovs.11-8194 PMC3208190

[10] Mak P, Leav I, Pursell B, Bae D, Yang X, et al (2010) ERbeta impedes prostate cancer EMT by destabilizing HIF-1alpha and inhibiting VEGF-mediated snail nuclear localization: implications for Gleason grading. Cancer Cell 17: 319–332. 20385358 10.1016/j.ccr.2010.02.030 PMC2881822

[11] Li D, Zhang C, Song F, Lubenec I, Tian Y, et al (2009) VEGF regulates FGF-2 and TGF-beta1 expression in injury endothelial cells and mediates smooth muscle cells proliferation and migration. Microvasc Res 77: 134–142. 18948122 10.1016/j.mvr.2008.09.007

[12] Lee KS, Park SJ, Kim SR, Min KH, Lee KY, et al (2008) Inhibition of VEGF blocks TGF-beta1 production through a PI3K/Akt signalling pathway. Eur Respair J 31: 523–531. 10.1183/09031936.00125007 18057050

[13] Tandon A, Tovey JC, Sharma A, Gupta R, Mohan RR (2010) Role of transforming growth factor Beta in corneal function, biology and pathology. Curr Mol Med 10: 565–578. 20642439 10.2174/1566524011009060565 PMC3048459

[14] Mandell KJ, Berglin L, Severson EA, Edelhauser HF, Parkos CA (2007) Expression of JAM-A in the human corneal endothelium and retinal pigment epithelium: localization and evidence for role in barrier function. Invest Ophthalmol Vis Sci 48: 3928–3936. 17724169 10.1167/iovs.06-1536 PMC2074894

[15] Cera MR, Fabbri M, Molendini C, Corada M, Orsenigo F, et al (2009) JAM-A promotes neutrophil chemotaxis by controlling integrin internalization and recycling. J Cell Sci 122: 268–277. 19118219 10.1242/jcs.037127

[16] Cooke VG, Naik MU, Naik UP (2006) Fibroblast growth factor-2 failed to induce angiogenesis in junctional adhesion molecule-A-deficient mice. Arterioscler Thromb Vasc Biol 26: 2005–2011. 16809549 10.1161/01.ATV.0000234923.79173.99

[17] Naik MU, Vuppalanchi D, Naik UP (2003) Essential role of junctional adhesion molecule-1 in basic fibroblast growth factor-induced endothelial cell migration. Arterioscler Thromb Vasc Biol 23: 2165–2171. 12958043 10.1161/01.ATV.0000093982.84451.87

[18] Naik MU, Naik UP (2006) Junctional adhesion molecule-A-induced endothelial cell migration on vitronectin is integrin alpha v beta 3 specific. J Cell Sci 119: 490–499. 16418218 10.1242/jcs.02771

[19] Samolov B, Steen B, Seregard S, van der Ploeg I, Montan P, et al (2005) Delayed inflammation-associated corneal neovascularization in MMP-2-deficient mice. Exp Eye Res 80: 159–166. 15670794 10.1016/j.exer.2004.08.023

[20] Reed NA, Oh DJ, Czymmek KJ, Duncan MK (2001) An immunohistochemical method for the detection of proteins in the vertebrate lens. J Immunol Methods 253: 243–252. 11384685 10.1016/s0022-1759(01)00374-x

[21] Kang LI, Wang Y, Suckow AT, Czymmek KJ, Cooke VG, et al (2007) Deletion of JAM-A causes morphological defects in the corneal epithelium. Int J Biochem Cell Biol 39: 576–585. 17118692 10.1016/j.biocel.2006.10.016

[22] Pal-Ghosh S, Blanco T, Tadvalkar G, Pajoohesh-Ganji A, Parthasarathy A, et al (2011) MMP9 cleavage of the beta4 integrin ectodomain leads to recurrent epithelial erosions in mice. J Cell Sci 124: 2666–2675. 21750188 10.1242/jcs.085480 PMC3138707

[23] Lin TC, Li CY, Tsai CS, Ku CH, Wu CT, et al (2005) Neutrophil-mediated secretion and activation of matrix metalloproteinase-9 during cardiac surgery with cardiopulmonary bypass. Anesth Analg 100: 1554–1560. 15920174 10.1213/01.ANE.0000154307.92060.84

[24] Russell RE, Culpitt SV, DeMatos C, Donnelly L, Smith M, et al (2002) Release and activity of matrix metalloproteinase-9 and tissue inhibitor of metalloproteinase-1 by alveolar macrophages from patients with chronic obstructive pulmonary disease. Am J Respir Cell Mol Biol 26: 602–609. 11970913 10.1165/ajrcmb.26.5.4685

[25] Hosseini H, Nejabat M, Mehryar M, Yazdchi T, Sedaghat A, et al (2007) Bevacizumab inhibits corneal neovascularization in an alkali burn induced model of corneal angiogenesis. Clin Experiment Ophthalmol 35: 745–748. 17997779 10.1111/j.1442-9071.2007.01572.x

[26] Matsuda A, Yoshiki A, Tagawa Y, Matsuda H, Kusakabe M (1999) Corneal wound healing in tenascin knockout mouse. Invest Ophthalmol Vis Sci 40: 1071–1080. 10235540

[27] Orosz ZZ, Katona E, Facsko A, Modis L, Muszbek L, et al (2011) Factor XIII subunits in human tears; their highly elevated levels following penetrating keratoplasty. Clin Chim Acta 412: 271–276. 20974119 10.1016/j.cca.2010.10.017

[28] Myrna KE, Mendonsa R, Russell P, Pot SA, Liliensiek SJ, et al (2012) Substratum topography modulates corneal fibroblast to myofibroblast transformation. Invest Ophthalmol Vis Sci 53: 811–816. 22232431 10.1167/iovs.11-7982 PMC3317421

[29] Gan L, Fagerholm P, Palmblad J (2004) Vascular endothelial growth factor (VEGF) and its receptor VEGFR-2 in the regulation of corneal neovascularization and wound healing. Acta Ophthalmol Scand 82: 557–563. 15453853 10.1111/j.1600-0420.2004.00312.x

[30] Sakimoto T, Sugaya S, Ishimori A, Sawa M (2012) Anti-inflammatory effect of IL-6 receptor blockade in corneal alkali burn. Exp Eye Res 97: 98–104. 22551515 10.1016/j.exer.2012.02.015

[31] Nupponen I AS, Järvenpää AL, Kautiainen H, Repo H. (2001) Neutrophil CD11b Expression and Circulating Interleukin-8 as Diagnostic Markers for Early-Onset Neonatal Sepsis. Pediatrics 108. 10.1542/peds.108.1.e12 11433091

[32] Jester JV, Petroll WM, Cavanagh HD (1999) Corneal stromal wound healing in refractive surgery: the role of myofibroblasts. Prog Retin Eye Res 18: 311–356. 10192516 10.1016/s1350-9462(98)00021-4

[33] Franco M, Roswall P, Cortez E, Hanahan D, Pietras K (2011) Pericytes promote endothelial cell survival through induction of autocrine VEGF-A signaling and Bcl-w expression. Blood 118: 2906–2917. 21778339 10.1182/blood-2011-01-331694 PMC3172806

[34] Lim JY, Oh MA, Kim WH, Sohn HY, Park SI (2012) AMP-activated protein kinase inhibits TGF-beta-induced fibrogenic responses of hepatic stellate cells by targeting transcriptional coactivator p300. J Cell Physiol 227: 1081–1089. 21567395 10.1002/jcp.22824

[35] Moldovan NI, Goldschmidt-Clermont PJ, Parker-Thornburg J, Shapiro SD, Kolattukudy PE (2000) Contribution of monocytes/macrophages to compensatory neovascularization: the drilling of metalloelastase-positive tunnels in ischemic myocardium. Circ Res 87: 378–384. 10969035 10.1161/01.res.87.5.378

[36] Perez-Santonja JJ, Campos-Mollo E, Lledo-Riquelme M, Javaloy J, Alio JL (2010) Inhibition of corneal neovascularization by topical bevacizumab (Anti-VEGF) and Sunitinib (Anti-VEGF and Anti-PDGF) in an animal model. Am J Ophthalmol 150: 519–528 e511. 20591397 10.1016/j.ajo.2010.04.024

[37] Aramwit P, Kanokpanont S, De-Eknamkul W, Srichana T (2009) Monitoring of inflammatory mediators induced by silk sericin. J Biosci Bioeng 107: 556–561. 19393558 10.1016/j.jbiosc.2008.12.012

[38] Li Z, Van Bergen T, Van de Veire S, Van de Vel I, Moreau H, et al (2009) Inhibition of vascular endothelial growth factor reduces scar formation after glaucoma filtration surgery. Invest Ophthalmol Vis Sci 50: 5217–5225. 19474408 10.1167/iovs.08-2662

[39] Cursiefen C, Chen L, Borges LP, Jackson D, Cao J, et al (2004) VEGF-A stimulates lymphangiogenesis and hemangiogenesis in inflammatory neovascularization via macrophage recruitment. J Clin Invest 113: 1040–1050. 15057311 10.1172/JCI20465 PMC379325

[40] Ambati BK, Nozaki M, Singh N, Takeda A, Jani PD, et al (2006) Corneal avascularity is due to soluble VEGF receptor-1. Nature 443: 993–997. 17051153 10.1038/nature05249 PMC2656128

[41] Vosseler S, Mirancea N, Bohlen P, Mueller MM, Fusenig NE (2005) Angiogenesis inhibition by vascular endothelial growth factor receptor-2 blockade reduces stromal matrix metalloproteinase expression, normalizes stromal tissue, and reverts epithelial tumor phenotype in surface heterotransplants. Cancer Res 65: 1294–1305. 15735015 10.1158/0008-5472.CAN-03-3986

[42] Shi L, Chang Y, Yang Y, Zhang Y, Yu FS, et al (2012) Activation of JNK signaling mediates connective tissue growth factor expression and scar formation in corneal wound healing. PLoS One 7: e32128. 22363806 10.1371/journal.pone.0032128 PMC3283717

[43] Bystrom B, Virtanen I, Rousselle P, Miyazaki K, Linden C, et al (2007) Laminins in normal, keratoconus, bullous keratopathy and scarred human corneas. Histochem Cell Biol 127: 657–667. 17492460 10.1007/s00418-007-0288-4

[44] West-Mays JA, Dwivedi DJ (2006) The keratocyte: corneal stromal cell with variable repair phenotypes. Int J Biochem Cell Biol 38: 1625–1631. 16675284 10.1016/j.biocel.2006.03.010 PMC2505273

[45] Mochida Y, Parisuthiman D, Pornprasertsuk-Damrongsri S, Atsawasuwan P, Sricholpech M, et al (2009) Decorin modulates collagen matrix assembly and mineralization. Matrix Biol 28: 44–52. 19049867 10.1016/j.matbio.2008.11.003 PMC2650102

[46] Vial C GJ, Santander C, Cabrera D, Brandan E (2011) Decorin interacts with connective tissue growth factor (CTGF)/CCN2 by LRR12 inhibiting its biological activity. J Biol Chem 286: 24242–24252. 21454550 10.1074/jbc.M110.189365 PMC3129205

[47] Zhang C, Tan CK, McFarlane C, Sharma M, Tan NS, et al (2012) Myostatin-null mice exhibit delayed skin wound healing through the blockade of transforming growth factor-β signaling by decorin. Am J Physiol Cell Physiol 302: 1213–1225. 10.1152/ajpcell.00179.2011 22277753

[48] Mohan RR, Tovey JC, Gupta R, Sharma A, Tandon A (2011) Decorin biology, expression, function and therapy in the cornea. Curr Mol Med 11: 110–128. 21342131 10.2174/156652411794859241

[49] Bates DO, Jones RO (2003) The role of vascular endothelial growth factor in wound healing. Int J Low Extrem Wounds 2: 107–120. 15866835 10.1177/1534734603256626

[50] Stalmans I, Vandewalle E, Van Bergen T (2010) [Vascular endothelial growth factor (VEGF) and modulation of wound healing after glaucoma surgery]. Verh K Acad Geneeskd Belg 72: 41–53. 20726439

[51] Shibuya M (2011) Vascular Endothelial Growth Factor (VEGF) and Its Receptor (VEGFR) Signaling in Angiogenesis: A Crucial Target for Anti- and Pro-Angiogenic Therapies. Genes Cancer 2: 1097–1105. 22866201 10.1177/1947601911423031 PMC3411125

[52] Zhang Z, Neiva KG, Lingen MW, Ellis LM, Nor JE (2010) VEGF-dependent tumor angiogenesis requires inverse and reciprocal regulation of VEGFR1 and VEGFR2. Cell Death Differ 17: 499–512. 19834490 10.1038/cdd.2009.152 PMC2822115

[53] Christoffersson G, Vågesjö E, Vandooren J, Lidén M, Massena S, et al (2012) VEGF-A recruits a proangiogenic MMP-9-delivering neutrophil subset that induces angiogenesis in transplanted hypoxic tissue. Blood 10.1182/blood-2012-04-421040 PMC3512240 22966168

[54] Burgu B, McCarthy LS, Shah V, Long DA, Wilcox DT, et al (2006) Vascular endothelial growth factor stimulates embryonic urinary bladder development in organ culture. BJU Int 98: 217–225. 16831171 10.1111/j.1464-410X.2006.06215.x

[55] Seghezzi G, Patel S, Ren CJ, Gualandris A, Pintucci G, et al (1998) Fibroblast growth factor-2 (FGF-2) induces vascular endothelial growth factor (VEGF) expression in the endothelial cells of forming capillaries: an autocrine mechanism contributing to angiogenesis. J Cell Biol 141: 1659–1673. 9647657 10.1083/jcb.141.7.1659 PMC2132998

[56] Murakami M, Nguyen LT, Hatanaka K, Schachterle W, Chen PY, et al (2011) FGF-dependent regulation of VEGF receptor 2 expression in mice. J Clin Invest 121: 2668–2678. 21633168 10.1172/JCI44762 PMC3223828

[57] Naik MU, Mousa SA, Parkos CA, Naik UP (2003) Signaling through JAM-1 and alphavbeta3 is required for the angiogenic action of bFGF: dissociation of the JAM-1 and alphavbeta3 complex. Blood 102: 2108–2114. 12750158 10.1182/blood-2003-04-1114

[58] Ford KM, Saint-Geniez M, Walshe TE, D'Amore PA (2012) Expression and Role of VEGF-A in the Ciliary Body. Invest Ophthalmol Vis Sci 53: 7520–7527. 23081980 10.1167/iovs.12-10098 PMC3493183

