# Supplementary figures and images for: Junctional Adhesion Molecule-A Regulates Vascular Endothelial Growth Factor Receptor-2 Signaling-Dependent Mouse Corneal Wound Healing
Source: PLoS One. 2013 May 8;8(5):e63674. doi: 10.1371/journal.pone.0063674 (PMC3648504; doi:10.1371/journal.pone.0063674)

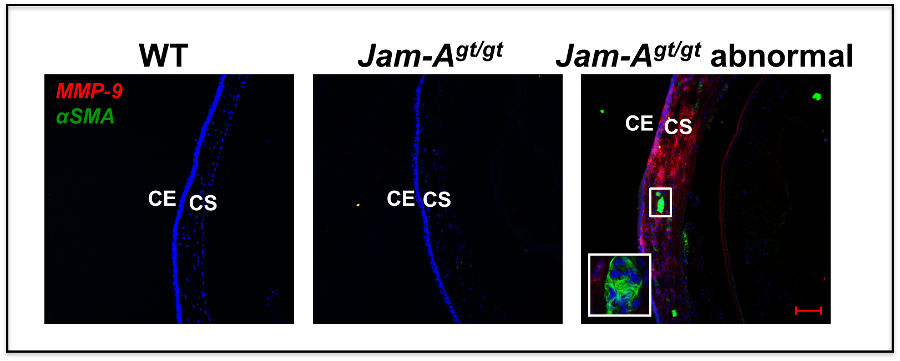

Supplement: Figure S1 — Source of MMP-9 expression in the cornea Jam-Agt/gt mice is not from αSMA expressing cells. Confocal images of co-immunostaining with MMP-9 and αSMA antibodies in WT, Jam-Agt/gt and Jam-Agt/gt abnormal eyes. Scale bar 100 µm. Inset magnifications are 10x of the highlighted region. Nuclear staining using Draq5 is shown in blue. CE: Corneal epithelium; CS: Corneal stroma. (TIF) [file pone.0063674.s001.tif]

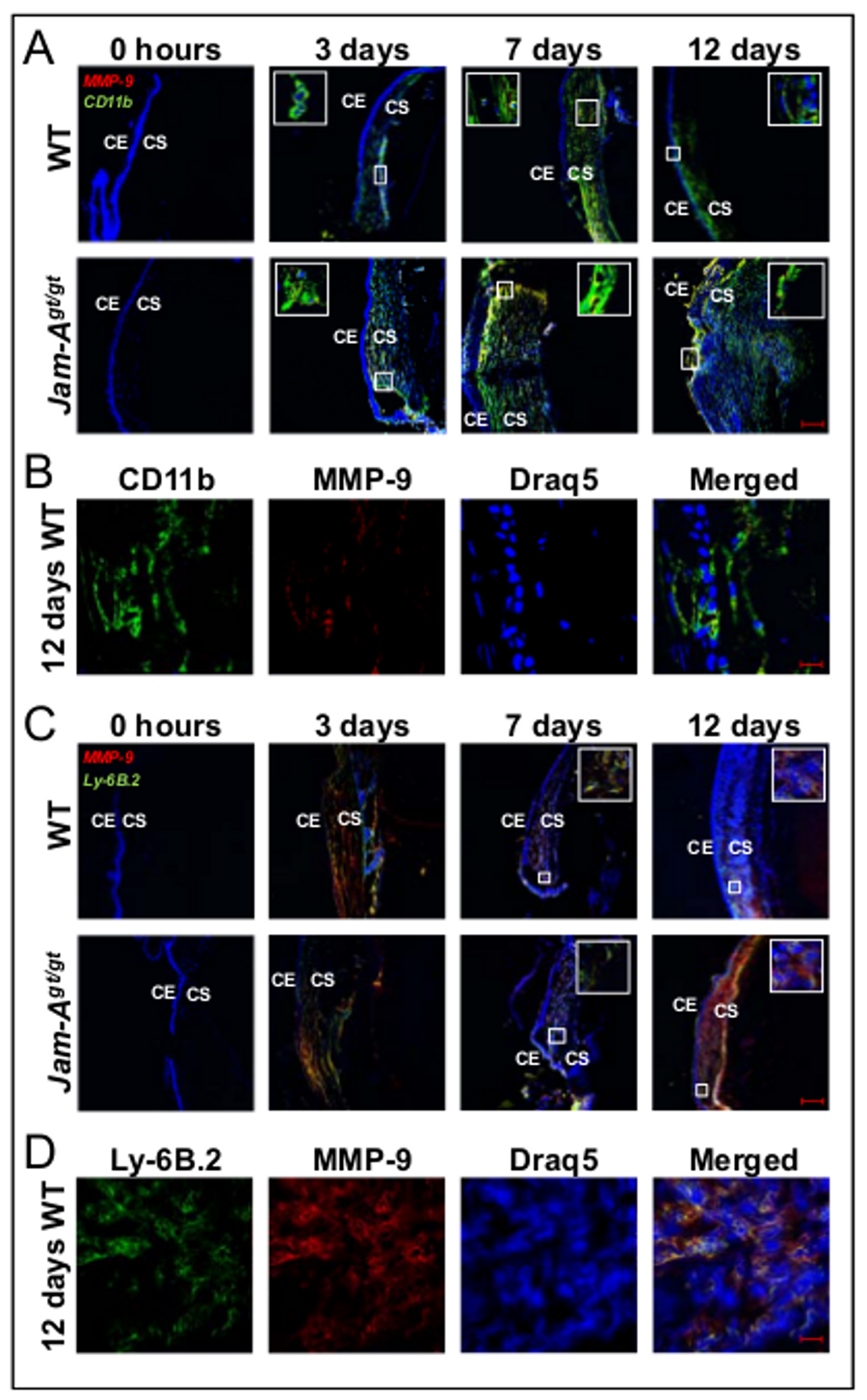

Supplement: Figure S2 — Inflammation observed in full thickness wounds is caused by neutrophils. (A–D) Confocal images of WT and Jam-Agt/gt eye sections. (A) Co-immunostaining with MMP-9 and CD11b antibodies show complete co-localization. Scale bar 100 µm. (B) Split representative images of MMP-9 and CD11b staining of 12 day WT eyes are also shown. Scale bar 10 µm (C) Co-localization of MMP-9 and Ly-6B.2 depicted in WT and Jam-Agt/gt corneas of full thickness injury eyes. Scale bar 100 µm. (D) Split images of MMP-9 and Ly-6B.2 staining of 12 day WT are also shown. Scale bar 10 µm. All inset magnifications are 10x of the highlighted region. Nuclear staining using Draq5 is shown in blue. CE: Corneal epithelium; CS: Corneal stroma. (TIF) [file pone.0063674.s002.tif]

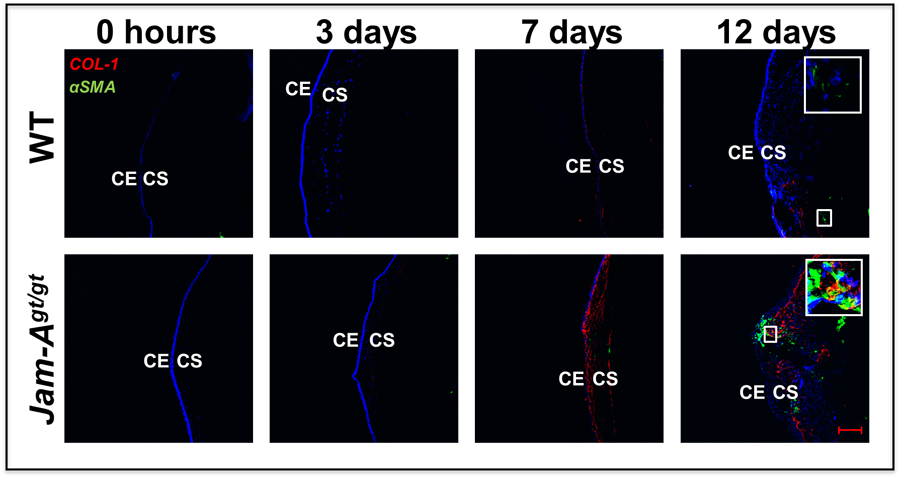

Supplement: Figure S3 — Full thickness Jam-Agt/gt eyes reveal fibrosis. Collagen I and αSMA expression indicative of fibrosis depicted in full thickness WT and Jam-Agt/gt corneas. Scale bar 100 µm. Inset magnifications are 10x of the highlighted region. Nuclear staining using Draq5 is shown in blue. CE: Corneal epithelium; CS: Corneal stroma. (TIF) [file pone.0063674.s003.tif]

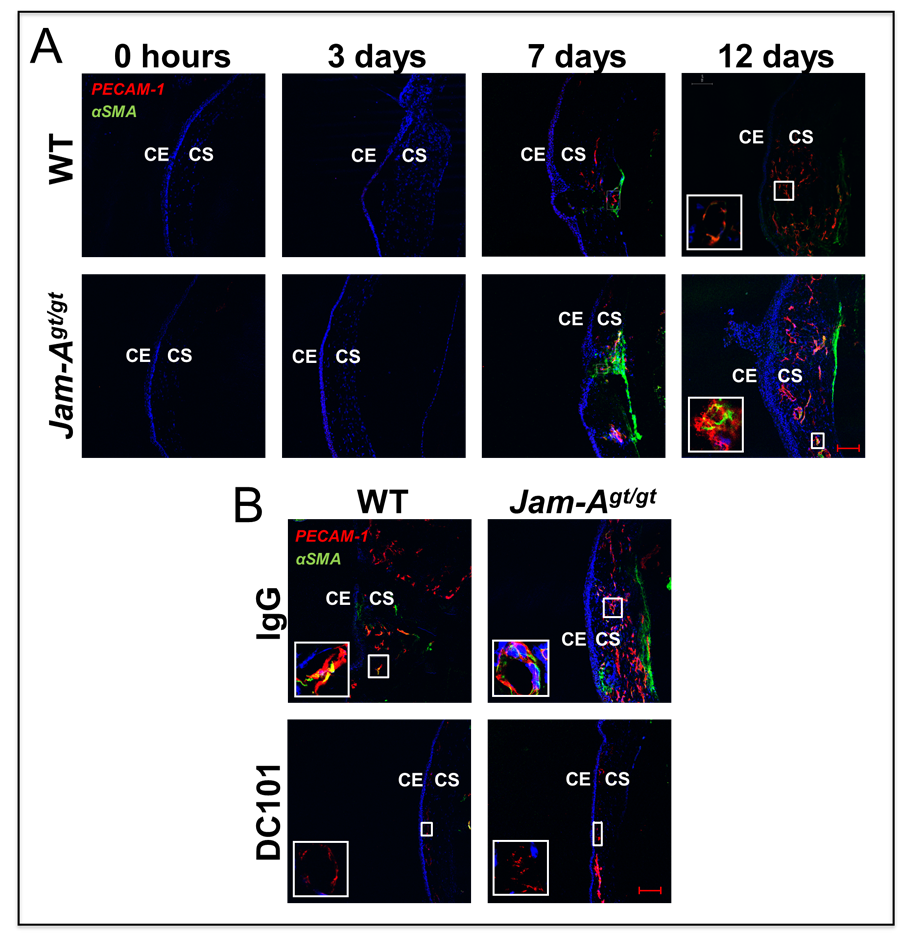

Supplement: Figure S4 — Pericyetes are not the major source of αSMA in full thickness wounds. (A) Full thickness wounds show minimal co-localization between PECAM-1 and αSMA staining. (B) Confocal images of co-immunostaining with PECAM-1 and αSMA antibodies in DC101 and IgG treated full thickness injury corneas. Scale bar 100 µm. All inset magnifications are 10x of the highlighted region. Nuclear staining using Draq5 is shown in blue. CE: Corneal epithelium; CS: Corneal stroma. (TIF) [file pone.0063674.s004.tif]

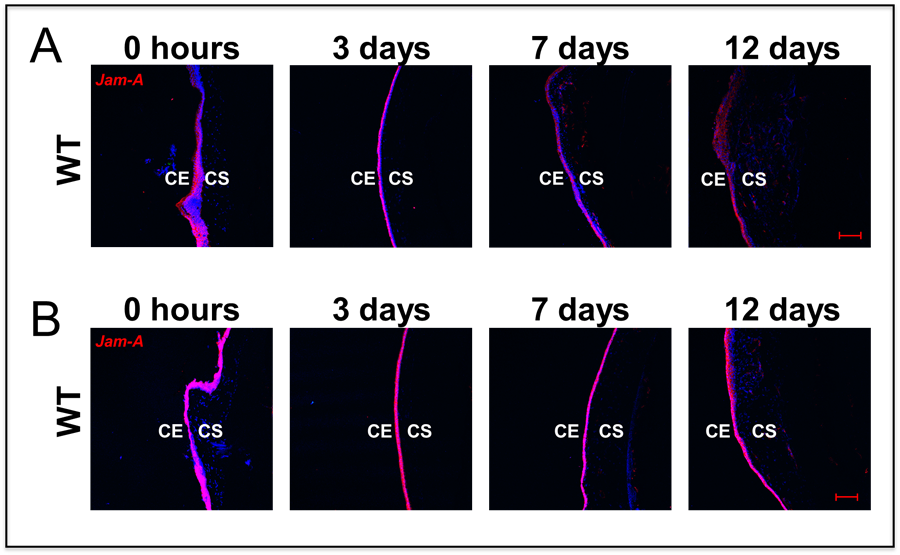

Supplement: Figure S5 — No difference in Jam-A expression in WT eyes during wound healing. (A) Jam-A staining of WT full thickness injury corneas at different time points post surgery. (B) Jam-A staining of WT silk sutured corneas. Scale bar 100 µm. Nuclear staining using Draq5 is shown in blue. CE: Corneal epithelium; CS: Corneal stroma. (TIF) [file pone.0063674.s005.tif]

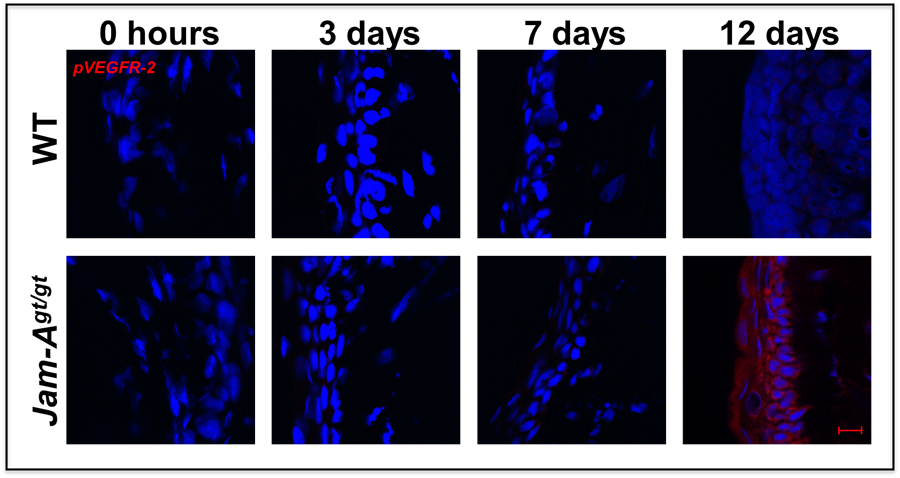

Supplement: Figure S6 — Full thickness wounds show VEGFR-2 activation in Jam-Agt/gt corneas. pVEGFR-2 staining in Jam-Agt/gt and WT eyes in full thickness injury model. Scale bar 10 µm. Nuclear staining using Draq5 is shown in blue. CE: Corneal epithelium; CS: Corneal stroma. (TIF) [file pone.0063674.s006.tif]
